# Treatment of Cutaneous Leishmaniasis with Sodium Stibogluconate and Allopurinol in a Routine Setting in Ethiopia: Clinical and Patient-Reported Outcomes and Operational Challenges

**DOI:** 10.3390/tropicalmed8080414

**Published:** 2023-08-14

**Authors:** Saskia van Henten, Fentaw Bialfew, Seid Hassen, Feleke Tilahun, Johan van Griensven, Seid Getahun Abdela

**Affiliations:** 1 Institute of Tropical Medicine, 2000 Antwerp, Belgium; jvangriensven@itg.be; 2 Boru Meda Hospital, Dessie P.O. Box 70, Ethiopia; 3 Department of Internal Medicine, Wollo University, Dessie P.O. Box 1145, Ethiopia; seidgech014@gmail.com

**Keywords:** *Leishmania aethiopica*, operational research, pentavalent antimonials, treatment extension

## Abstract

Cutaneous leishmaniasis (CL) is common in Ethiopia, but the national guideline does not offer specific treatment recommendations. Consequently, different treatment regimens are used in the country, without quality evidence. In Boru Meda Hospital, sodium stibogluconate (SSG) is routinely used in combination with allopurinol for systemic CL treatment, although evidence on its effectiveness is limited. An observational cohort study was carried out to document clinical treatment outcomes in patients receiving SSG/allopurinol at the end of each 28-day treatment cycle and after 180 days. Patient-reported outcomes were assessed by asking patients to rate lesion severity, and by the dermatological life quality index. A total of 104 patients were included. After one treatment cycle, only four patients were clinically cured, although patient-reported outcomes significantly improved. The majority (88) of patients were appointed for a second treatment cycle, of whom only 37 (42%) attended. Among the 36 patients who came for final outcome assessment, 50% were cured. Follow-up and treatment were severely affected by conflict; drug stock-outs and insufficient ward capacity for treatment were additional challenges. The treatment outcomes of SSG/allopurinol were relatively poor, and most patients required more than one cycle of treatment. Shortages of drugs and beds indicate the existing gaps in providing CL treatment in Ethiopia.

## 1. Introduction

Cutaneous leishmaniasis (CL) is a common dermatological condition in Ethiopia, where approximately 20,000–50,000 patients are affected each year [1]. It is mainly caused by *Leishmania aethiopica*, which is a unique species that typically causes severe lesions on the face that are difficult to treat [2].

Due to the lack of good-quality evidence for *L. aethiopica* treatment outcomes, the national guideline does not recommend specific treatments [3] but only mentions therapeutic options without specifying the duration. In practice, treatment extension is common [4,5,6], and different combinations of treatment regimens are practiced [4,6,7]. As treatment efficacy is species-dependent, there is a dire need to document the outcomes of routinely used treatment options for *L. aethiopica*. Intravenous or intramuscular sodium stibogluconate (SSG) remains the most widely used treatment in Ethiopia. In Boru Meda General Hospital, treatment with systemic SSG is routinely combined with low-dose allopurinol, but outcomes have never been reported.

Looking beyond clinician-assessed cure rates, accounting for patients’ perspectives is crucial, especially for diseases that negatively affect individuals through psychosocial impact. A recent publication [8] called for including outcomes related to quality of life in studies looking at treatment effectiveness. To date, this has rarely been done around the globe.

In Ethiopia, anecdotal evidence also points to operational challenges such as drug shortages, insufficient bed capacity for inpatient treatment, and poor patient follow-up. Better documentation of these challenges is needed to properly outline and address the current gaps in CL treatment. Additionally, parasitological confirmation through demonstration of parasites is required according to the national guideline, while monitoring of renal and hepatic function as well as electrocardiography changes is mentioned to evaluate toxicity, without any mention of specific frequency. However, it is unclear how often this is performed routinely.

In this study, we wanted to document SSG/allopurinol clinical treatment outcomes, determine how often treatment extension was given, and explore how patients reported their outcomes by looking at patient-reported outcomes using the dermatological life quality index, as well as patient-rated lesion severity. We also described the operational challenges encountered throughout this project.

## 2. Materials and Methods

### 2.1. Setting

The research took place at Boru Meda General Hospital. This hospital provides specialized dermatology and ophthalmology services, with a high caseload of leprosy and CL patients [9]. There is a dermatology ward that has 40 beds. Patients who are confirmed either clinically or microscopically with a skin slit smear are treated either with local treatment by intralesional SSG or cryotherapy (if available), or with systemic treatment.

Routine treatment for severe CL cases that need systemic treatment consists of one or more cycles of 28 days of intramuscular 20 mg/kg SSG, with a maximum dose of 850 mg/day, in combination with one 100 mg tablet of allopurinol. SSG is given intravenously in case the patients cannot tolerate intramuscular injections. Patients are typically admitted for the entire course of treatment. SSG is made available through the World Health Organization via the Ministry of Health.

### 2.2. Design, Population, Recruitment, and Sample Size

This was an observational cohort study among patients with CL who received systemic SSG with allopurinol following routine clinical care decisions. Patients with a clinical or parasitological diagnosis of CL who were started on SSG with allopurinol were invited to the study consecutively. Study recruitment started on 15 February 2021, with the last patient recruited on 4 August 2022, with several interruptions due to political unrest in the area from 3 November 2020 until 3 November 2022.

Patients’ visits were planned at baseline (BL) before starting treatment, for each subsequent treatment cycle (cycle 1 (C1), cycle 2 (C2), etc.), with study visits planned at the end of each treatment cycle before discharge, as well as at day 180 (D180) after starting treatment. Patients were also instructed to come back between visits if their lesion did not respond to treatment or was worsening. All information produced during routine diagnosis and treatment was captured, including lab tests that were performed and their respective results.

The original sample size was planned to be 117 patients. Sample size calculation was based on the proportion of cure at D180, with a required precision of 10%, using a 95% confidence level and a conservative estimate of 50% cure. This gave a sample size of 97 patients, which was inflated by 20% to account for loss to follow-up, leading to a final sample size of 117 patients. Due to the conflict and stock-outs, recruitment took much longer than planned. Therefore, recruitment was terminated prematurely after a long treatment stock-out prevented the inclusion of individuals beyond 104 patients.

### 2.3. Lesion Assessment

Before treatment started, the study clinician assessed the lesion by measuring the size of the largest diameter, counting the number of active lesions, and noting the lesion characteristics (nodular, plaque, ulcerated etc.). Patients were classified as localized CL (LCL), mucocutaneous CL (MCL), and diffuse CL (DCL) by the treating clinician. Although no clear-cut definitions for these types exist, broadly, MCL involves the mucosa or the mucosal border, DCL affects multiple body parts, and LCL lesions are limited and only affect the skin [3]. The largest lesion was considered as the index lesion.

### 2.4. Patient-Reported Outcome Measures

The Amharic versions of the dermatological life quality index (DLQI, which was previously validated for podoconiosis patients in southern Ethiopia [10]) and children’s DLQI (cDLQI) questionnaires [11] (for those aged 4–16 years) were administered to patients by the study staff at each study visit (BL, C1, C2 (if applicable), etc., D180). The DLQI questions were not asked to patients below the age of four years. For children up to 8 years old, the questions were posed to the parent/guardian, while for those aged 8 to 12 years, the questions were asked both to the patient and the parent/guardian. For children above 12 years old, only the child’s answers were considered. The questionnaires were scored and analyzed as recommended [11,12], with total scores categorized from no effect to extremely large effect, and removing questionnaires with answers on eight or less questions as invalid.

Patient-reported outcomes were assessed by asking patients to rate the severity of their lesion on a Likert scale as clear (1), almost clear (2), mild (3), moderate (4), or severe (5) at BL, C1 and any subsequent cycles, and D180.

### 2.5. Clinical Outcome Assessment

Lesions were assessed by the clinician at the end of each treatment cycle and at D180, according to slightly adapted internationally used criteria and cutoffs for outcome definitions [12]. Patients were classified as cured if all lesions that were present at BL had complete reepithelization (if ulcerated) and flattening. Patients were categorized as substantial improvement if not all lesions were cured but there was at least 50% improvement of all lesions compared to BL. Minor improvement required all lesions to have at least 1–49% improvement compared to BL. Worsening was classified compared to the previous lesion assessment, which could be either due to worsening of previously present lesions, or the appearance of new lesions. Photographs of the lesions were taken at each study visit to capture the lesion characteristics and outcomes for cross-checking and quality control.

Although we initially planned to include only outcomes 120–240 days after starting treatment, due to the conflict and subsequent travel restrictions, we were more lenient and considered all outcome assessment more than 110 days after starting treatment for the D180 outcome.

### 2.6. Sample Collection

This study planned to use the routine skin slit for PCR confirmation, and we collected blood and non-invasive tape disc samples (D-Squame, MonaDerm) from each study visit for further analysis.

### 2.7. Data Collection and Analysis

A paper data collection form was used to collect patient information, which was double-entered into a REDCap database [13]. Analysis was conducted using R version 4.1.3 [14]. To describe the population, numbers and proportions with medians and interquartile ranges (IQRs) were used. Treatment outcomes were analyzed as categorical variables with multinomial 95% confidence intervals (95% CIs). The cure rate was also analyzed as a proportion, with binomial 95% CIs. Differences in DLQI scores and patient global assessment over time were analyzed using the Wilcoxon signed-rank test for paired data, while the Mann–Whitney test was used to compare scores between groups. Kappa coefficients were used to measure agreement between when patients rated their lesion as clear and when the clinician rated the lesion as cured.

### 2.8. Patient Tracing

Patient tracing was carried out over the phone, using patient information (i.e., phone number of the patient and at least one family member/neighbor) collected at BL. Patients were called if they were late for an appointment to ask them if they wanted to come. At the end of the study, all patients who missed their D180 visit were called to ask whether they thought their lesion was cured, substantially improved, the same, or worse compared to before, and they were asked why they missed their outcome visit.

## 3. Results

A total of 104 patients were included in the study (Supplementary Figure S1), as described in Table 1. The majority (85; 81.7%) were referred from health centers or primary hospitals. Patients frequently (74/104; 71.1%) used traditional treatment before coming to the hospital for CL care. Many patients had also tried several modern medications such as ointments and (mostly systemic) antibiotics, but only six (5.8%) had previously been treated for CL at a known CL treatment center.

The median duration of symptoms at enrollment into the study was 11.5 months (IQR 7.0–17.0). For three-quarters of the patients (80; 76.9%), the full cost of their admission and treatment was covered by health insurance. The patients came from districts up to 200 km away, although they more frequently came from areas close to the hospital.

**Table 1:** Baseline characteristics of included patients.

	Total N = 104
Age, median (years)	18.0 (11.8–30.0)
Sex, male	71 (68.3)
**CL** (**Treatment**) **history**	
Referred from other health facility	85 (81.7)
Previous CL episode (*n* = 103)	6 (5.8)
Traditional treatment used	74 (71.1)
Herbal	64 (61.5)
Holy water	12 (11.5)
Burning	1 (0.9)
Other	3 (2.9)
Modern treatment (any) ^a^	40 (38.5)
CL-specific treatment	6 (5.8)
**Lesion characteristics**	
Duration in months, median (IQR)	11.5 (7.0–17.0)
Type of lesion	
LCL	30 (28.8)
MCL	69 (66.3)
DCL	5 (4.8)
No. of active lesions	
1	70 (67.3)
2	16 (15.4)
3	10 (9.6)
≥4	8 (7.7)
Presence of concomitant CL scar	5 (4.8)
Size (largest diameter), median IQR (*n* = 100)	5.0 (4.0–8.0)
Location of index lesion	
Face	90 (86.5)
Arm/hand	10 (9.6)
Other body part	4 (3.8)
Index lesion presentation	
Plaque	98 (94.2)
Scaly	62 (59.6)
Crusted	55 (52.9)
Papular	54 (51.9)
Erythematous	35 (33.7)
Hyperpigmented	31 (28.9)
Swollen	22 (21.2)
Nodular	21 (20.2)
Ulcerated	18 (17.3)

CL: cutaneous leishmaniasis, DCL: diffuse cutaneous leishmaniasis, IQR: interquartile range, LCL: localized cutaneous leishmaniasis, MCL: mucocutaneous leishmaniasis. ^a^ Any ointment, tablets, syrup, or injection received at a health post, health center, hospital, or pharmacy In cases of missing data, the total patients with available data are indicated with (*n* = x) behind the respective variable.

### 3.1. Patient Characteristics

**Figure 1:**
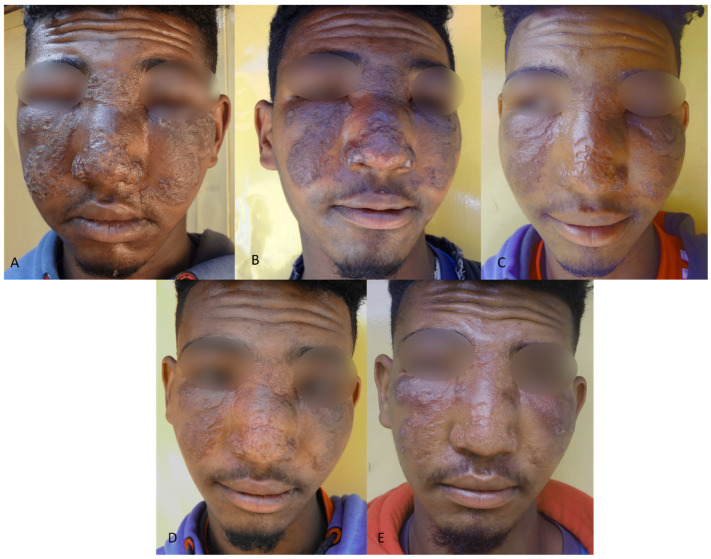
**A patient with extensive mucocutaneous leishmaniasis**: (**A**) Swollen, erythematous, and crusted plaques on the face which restricted opening of the eyelid. The patient rated his lesion as severe and had a DLQI score of 16. (**B**) Reduced swelling and crusting with flattening and darkening of the lesions after treatment cycle 1, with substantial improvement as the clinical outcome. DLQI score was 9 and patient-reported severity was moderate. (**C**) Further reduction in swelling and plaque formation, with substantial improvement as the clinical outcome after cycle 2. DLQI score 8 and patient assessment was mild. (**D**) Lesion was rated as cured with significant remaining scar tissue after cycle 3. Swelling around the eyes had almost completely gone. DLQI was 3 and the patient rated the lesion as mild. Treatment was not extended after the third cycle. (**E**) Worsening with increased swelling, erythema, and papules on both cheeks at D180. The patient rated his DLQI as 20, and his lesion as severe.

The median age was 18.0 years, with 25% (26) of patients younger than twelve years. More than half of patients (69; 66.3%) presented with MCL, whereas five (4.8%) were classified as DCL. A patient with extensive MCL is shown in Figure 1. Most patients had a single lesion, which was most frequently on the face.

### 3.2. Impact of CL before Starting Treatment

**Figure 2:**
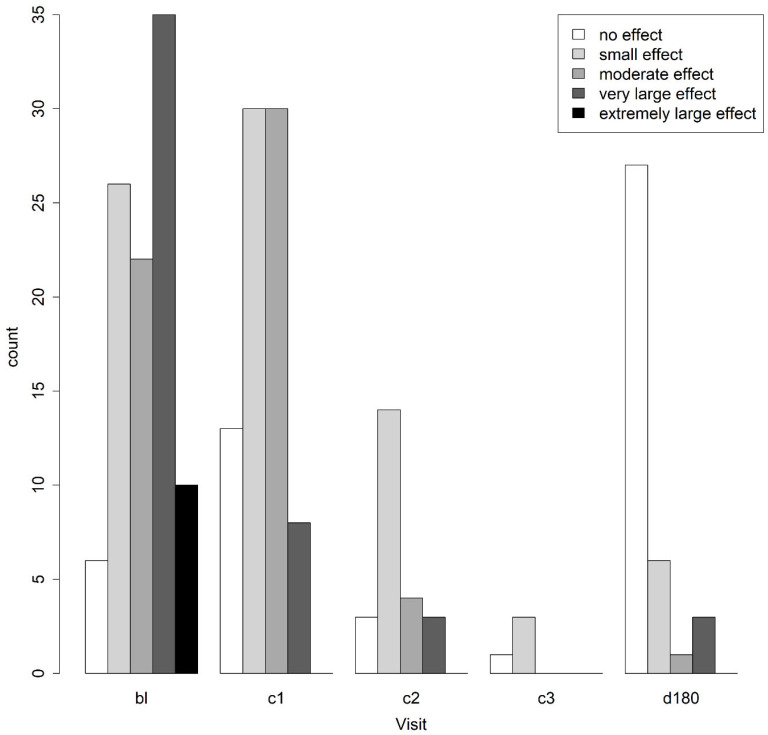
Dermatological life quality index results by study visit. The number of patients in each of the following categories is shown per study visit: no effect, small effect, moderate effect, very large effect, extremely large effect. Results for adults and for children aged 4–15 are combined in this figure. Bl: baseline, c1: after cycle 1, c2: after cycle 2, c3: after cycle 3, d180: after 180 days since starting treatment.

DLQI data were available for 99 patients before starting treatment, after excluding five invalid questionnaires. The data are shown overall in Figure 2 and separated by the questionnaire used (children when aged <15 years and adult when aged ≥15) in Supplementary Table S1. Overall, the median DLQI score was 10 (IQR 5–16). Six (6.1%) patients experienced no effect, twenty-six (26.3%) reported a small effect, twenty-two (22.2%) reported a moderate effect, thirty-five (35.4%) reported a very large effect, and ten (10.1%) reported an extremely large effect. The domain that was most affected for both children (Supplementary Table S2) and adults (Supplementary Table S3) was symptoms and feelings (median score 2.0 (1.0–3.0) for children and 3.0 (2.8–4.0) for adults). Adults suffered significantly (*p* < 0.001, Mann–Whitney test) more impact due to CL than children, with a median score of 12.5 (IQR 8.0–18.0) compared to 7.0 (IQR 3.0–11.0).

Using patient-reported severity (Table 2), more than half of patients (56; 53.8%) rated their lesions as severe before treatment, with another 38 (36.5%) rating their lesion as moderate.

**Table 2:** Patient-reported severity assessment at the different study visits.

Category	Baseline Total (%) N = 104	End of Cycle 1 (%) N = 89	End of Cycle 2 (%) N = 24	Day 180 N = 32 (30.8)
Clear	0 (0)	1 (1.1)	3 (12.5)	11 (34.4)
Almost clear	0 (0)	18 (20.7)	7 (29.2)	8 (25.0)
Mild	9 (8.7)	39 (44.8)	10 (41.7)	5 (15.6)
Moderate	38 (36.5)	28 (32.2)	4 (16.7)	4 (12.5)
Severe	56 (53.8)	1 (1.1)	0 (0)	4 (12.5)
*Missing* ^a^	*1/104 (1.0)*	*2/89 (2.2)*	*1/24* ^b^ *(4.2)*	*4 (11.1)*
Median	5.0 (4.0–5.0)	3.0 (3.0–4.0)	3.0 (2.0–3.0)	2.0 (1.0–3.3)

^a^ Missing proportions were calculated by taking the total number of patients who came for that visit. ^b^ Patient global assessment was taken from one patient for whom the clinical assessment was missed, but missed from one patient who had a clinical outcome.

### 3.3. Laboratory Testing

A skin slit smear test was performed to confirm CL at BL in almost all (99; 95.2%) patients, but it was negative in 56 (56.6%) patients. Although two additional patients were confirmed by fine-needle aspiration cytology, and two patients had a previous positive skin slit smear, the majority of the patients (57; 54.8%) were treated based on clinical diagnosis alone. Although complete blood counts (CBCs) and organ function tests were routinely requested before starting treatment, they were performed for only 39 (37.5%) and 24 (23.1%) patients, respectively, at the start of the first treatment cycle. HIV tests were carried out for a total of 22 patients (21.2%), and for only 1 of the 5 DCL patients; all patients tested were HIV-negative.

During the first cycle of treatment, CBC was recorded for half of the patients (53; 51.0%). Six (5.8%) patients had more than one CBC result. Blood counts were mostly tested in the middle of the treatment period. Organ function tests were performed in C1 for less than one-quarter of patients (24; 23.1%). An electrocardiogram (ECG) was done for only two patients.

### 3.4. Treatment and Follow-Up

A total of 99 patients completed their first treatment cycle of SSG/allopurinol combination treatment, of whom 89 completed all C1 study procedures (Supplementary Figure S1). Most missed C1 study visits took place during fall 2020, when the hospital was overloaded with soldiers from the front, and study staff were unable to perform their study activities alongside their routine engagements. Among the 88 patients appointed for retreatment at C2, only 37 (42.0%) actually came for a second treatment cycle during the study period (Supplementary Figure S1). One patient suddenly died in the middle of her second cycle, which could potentially have been related to cardiotoxicity from SSG, but since no ECG was performed this could not be confirmed. Three patients were discharged before finishing their C2 visit because the hospital was evacuated. For nine study patients, procedures for the C2 study visit were missed, mainly due to the conflict. Only around one-third (36/104; 34.6%) of patients came for their D180 outcome assessment.

### 3.5. Clinical Treatment Outcomes

The clinical outcomes are shown in Table 3. Among the 89 patients who completed the C1 study procedures, 4 (4.5%) were cured after 28 days, 71 (79.8%) had substantial improvement, 12 (13.5%) had minor improvement, 1 (1.2%) had no improvement, and another 1 (1.2%) was worsening. The majority (83/89; 92.8%) of the patients were appointed for another cycle of treatment (Supplementary Figure S1).

After finishing the second treatment cycle, the majority of patients with a known outcome (20/24; 83.3%) had substantial improvement; two (8.7%) were cured, and two (8.7%) had minor improvement (Table 3). For the majority of the treated patients (21/24; 87.5%), another treatment cycle was deemed necessary (Supplementary Figure S1).

Half of the patients who came for their D180 visit (18/36; 50.0%) were cured, 11 (30.6%) had substantial improvement, 2 (5.6%) had minor improvement, and 5 (13.9%) were worsening. Four study patients were continued on treatment after finishing their D180 visit. Outcomes were significantly better (*p* = 0.032) in the group who came according to the ordered treatment extension, with eight of ten cured at their D180 outcome, while this was ten out of twenty-five (40.0%) in those who did not come for planned treatment extension. Still, these ten patients who were cured, along with another ten who had substantial improvement, indicate that further self-healing of the lesion can still take place even if further treatment is deemed necessary. Treatment outcomes were not significantly different for patients with a confirmed diagnosis compared to those treated empirically (*p* = 0.088 for C1, *p* = 0.772 at C2, and *p* = 0.785 at D180; Fisher’s exact test).

**Table 3:** Clinical treatment outcomes at the different study visits.

Category	C1 (%) N = 89 (85.6)	95% CI	C2 (%) N = 24 (23.1)	95% CI	C3 (%) N = 4 (3.8)	D180 (%) N = 36 (34.6)	95% CI
Cure	4 (4.5)	0–13.1	2 (8.3)	0–24.7	2 (50.0)	18 (50.0)	36.1–68.4
Substantial improvement	71 (79.8)	73.0–88.3	20 (83.3)	75.0–99.7	2 (50.0)	11 (30.6)	16.7–48.9
Minor improvement	12 (13.5)	6.7–22.1	2 (8.3)	0–24.7		2 (5.6)	0–23.9
No improvement	1 (1.1)	0–9.7	0 (0)	0–16.4		0 (0)	0–18.3
Worsening	1 (1.1)	0–9.7	0 (0)	0–16.4		5 (13.9)	0–32.3
*Missing* ^a^	*10 (10.1)*		*9 (27.3)*		0	0	

^a^ Missing data were calculated from the total number of patients who completed the treatment cycle, which was 99 for C1, 33 for C2, and 4 for C3.

### 3.6. Patient-Reported Outcome Data

Patient-reported outcomes are shown in Table 2. After one cycle, patients rated their lesion severity as significantly improved compared to BL (*p* < 0.001, Wilcoxon signed-rank test), with a median score of 3.0 (IQR 3.0–4.0) compared to 5.0 (IQR 4.0–5.0) at BL.

At D180, around one-third (11/32; 34.4%) of patients reported their lesions to be cleared, one-quarter (8/32; 25.0%) said that they were almost cleared, five (15.6%) said that they were mild, four (12.5) assessed them to be moderate, and four (12.5) assessed them as severe. The median score given at D180 was significantly lower than at BL (*p* < 0.001, Wilcoxon signed-rank test).

Overall, the proportion of patients cured according to the clinician was significantly different from the patient-reported clearance rate (Table 4). The clinician cure rate was higher (13.0 vs. 8.3) at C2, but lower than patient-reported clearance rates at C1 (4.5 vs. 1.1) and D180 (50.0 vs. 34.4). Agreement as measured by the kappa coefficient between clinical cure and patient-reported cure was 0.69 at D180 and 0.78 at C2. At C1, this was much lower, at 0.38.

### 3.7. Dermatological Life Quality Index after Treatment

The results from the DLQI data are shown in Figure 2. Baseline and C1 data were available for 79 patients, whose median score was 9 (IQR 5–15) at BL and 6 (IQR 3–8) at C1. After treatment, the DLQI scores of the 79 patients with BL and C1 data decreased significantly (*p* < 0.001, Wilcoxon signed-rank test), with a median score of 6.0 (IQR 3.0–8.0) at the C1 study visit, compared to 9 (IQR 5–15) at BL. At D180, the median DLQI score of patients was 0 (IQR 0–2), with 27 (73.0%) patients having no effect, 6 (16.2%) having a small effect, 1 (2.7%) having a moderate effect, and 3 (8.1%) having a very large effect.

### 3.8. Tracing Patient Outcomes

Because so few patients came for the outcome assessment visit, we systematically called up all patients to ask how they perceived their lesion now, and why they did not come for their D180 visit.

We were able to reach three-quarters (52/67; 77.6%) of the missed patients by phone. Over half (29/52; 55.8%) of them reported that their lesion was cured, around one-third (17/52; 32.7%) said that there was improvement, and four patients (7.7%) reported no change or worsening. The most common reasons that the D180 visit was missed were because of the conflict (13/52; 25%), because the lesion was deemed to be cured already (12/52; 23.1%), and due to lack of money or time (10/52; 19.2%). Three patients reported that they did not come because further treatment was not available.

### 3.9. Operational Challenges

Many operational challenges were encountered during the study. Drug stock-outs occurred three times: allopurinol was unavailable for about one month in April 2021; SSG stock-outs occurred twice—once for six weeks from late March 2022 until the 8 May 2022, and for a second time from the 12 August 2022 until the end of the study (over 3.5 months), which also led to early termination of recruitment.

Due to conflict, the hospital became one of the main treatment sites for wounded soldiers. As the front advanced further south, the hospital staff were forced to evacuate on the 14 October 2021. During the months in which the hospital was not operational, the study freezer (containing all study samples) and camera were stolen, and several documents were lost. Hospital services were restarted from February 2022 onwards.

Another challenge was that the patients requiring treatment exceeded the ward capacity. In October 2022 there was a list of 171 patients waiting for admission for CL treatment. Of these, 24 were study patients.

## 4. Discussion

This paper reports on the routine use of systemic SSG in combination with allopurinol in Ethiopia. At the end of the first treatment cycle, only four patients were considered to have been cured, whereas almost all of the other patients were appointed for another treatment cycle. Less than half of the appointed patients returned for further treatment, and only a small number of patients came for their final outcome assessment visit, where around half of them were cured and around one-third had substantial improvement. According to their own assessment, one-third said that their lesion had cleared, but around 70% said that their lesion no longer had an impact on their quality of life. In addition, this study highlights the effects of the conflict in northern Ethiopia on treatment provision for CL, and it also shows that drug stock-outs and shortage of beds—problems not directly related to the conflict—impact treatment.

Most patients had insurance [15] that covered the cost of treatment, and since patients need to be referred in order to qualify for health insurance to cover hospital costs, the majority were referred from other health facilities. However, the fact that most patients still came with longstanding lesions after trying traditional treatments and various other modern treatments highlights delays in seeking care. This likely contributes to larger lesion size and more severe scar formation, thereby aggravating the psychosocial impact of CL.

In this patient population receiving systemic treatment, the impact of CL on their quality of life prior to treatment was high, with 35% of patients indicating a very large and 10% an extremely large impact. More than half of the patients rated their lesion(s) as severe before treatment. This clearly shows the great impact that CL has on these patients, and it emphasizes the need for treatment and psychosocial support. Similar findings were shown by Doni et al. in a recent preprint [16]. Although clinical cure rates at the final visit were relatively poor (around 50%), an additional 30% had substantial improvement, and patient-reported outcomes at this time indicated a significant improvement in their quality of life as well as their severity ratings of their lesion(s). Although social desirability bias may play a role, our findings seem to highlight that even though the proportion of patients cured was low, most patients experienced a significant improvement in their lesion appearance and associated quality of life. Therefore, the treatment still seemed to have a positive impact on patients.

In this setting, systemic SSG (20 mg per kg, with a maximum of 850 mg/day) is routinely used with low-dose allopurinol (100 mg per day), without a clear evidence base. Evidence on the use of allopurinol for treatment of CL is generally conflicting. Clinical trials in Iran [17] and Colombia [18,19] showed that allopurinol significantly increased the effectiveness of antimonials, and that this combination could cure lesions that failed to respond to other treatments [20,21,22]. On the other hand, randomized clinical trials in Peru [23] and Iran [24] showed no added benefit of allopurinol compared to antimonials alone. However, almost all previous studies used allopurinol at a high dose of 20 mg/kg, adding up to a daily dose that usually exceeded 1000 mg—in stark contrast to our study, where patients received a daily allopurinol dose of only 100 mg. This low dose was started based on expert opinion, without proper evidence, and has been continued since only at this center, further underlining the need for clear evidence-based treatment guidelines.

Due to the low number of patients with final treatment outcomes, we should be careful when drawing conclusions about the effectiveness of SSG/low-dose allopurinol. Poor follow-up data were partially caused by the conflict but also related to treatment outcomes, as both patients with very poor outcomes and those who are already cured are unlikely to come to follow-up. However, informal outcome data collected by calling patients indicated that in patients who missed their outcome visit, the proportions cured and improved were similar to those who attended their outcome visit. Cure rates were similar to what our group reported for 28 days of miltefosine treatment [25]. Since the majority of the cured patients in this study were hospitalized for at least two treatment cycles of SSG with allopurinol, miltefosine seems preferable, as patients took their treatment at home after one week of hospitalization.

The decision to extend treatment was very common, although many patients never actually returned for further treatment. It is hard to draw conclusions about the outcomes of treatment extension, as the lesion severity and response to the first treatment cycle were likely related to patients coming for follow-up. Since more than half of the patients who did not come for their appointed treatment extension were still cured at D180, treatment extension does not always seem to be needed. However, at Boru Meda Hospital, due to fear of relapse, treatment is typically extended if the lesion is not cured, which is assessed directly or two weeks after completing the first treatment cycle. Some argue that this is too early, as tissue repair is thought to still take several weeks after the parasites have been killed [26]. Formal randomized studies are needed to assess the additive effect of treatment extension, but consensus on when and for whom treatment extension is needed could also provide more clarity.

Our study highlights several important operational challenges of treating CL in Ethiopia. Drug stock-outs were frequently seen, indicating that drug supplies are not consistently reliable. SSG is mainly supplied by the WHO through the Ministry of Health, but it seems that the supply does not match the current needs of CL treatment centers. Although antileishmanial drugs are listed in the essential drug list of the country [27], consistent supply should be ensured. The impact of the conflict on CL treatment was large, as hospital services were shut down for months, treatments were interrupted, and follow-up was perturbed. Unavailability of drugs and interruption of services probably contributed to the long waiting list for inpatient treatment. Insufficient treatment capacity further interfered with treatment schedules and follow-up, as most patients who were reappointed for treatment did not come, or they could not be treated due to lack of space. Ambulatory treatment with oral miltefosine as an oral drug could solve part of the problem, although cost and access are issues that currently limit widespread implementation.

A recent study in Ethiopia highlighted that there is severe underreporting of CL, and that the majority of patients never seek care at modern treatment facilities [28]. According to the 2030 Neglected Tropical Disease (NTD) roadmap [29], 85% of all CL cases should be detected and 95% of them should be treated, which would increase patient flow to treatment centers. The long waitlist for inpatient treatment indicates that treatment facilities may not have the capacity needed to treat the CL caseload and meet the roadmap targets. To succeed, there should be a great scaling-up of referral treatment sites if inpatient treatment with systemic antimonials is to be continued. Boru Meda has recently upgraded its dermatological treatment capacity with 16 beds, but waiting lists for treatment persist. Instead, innovative approaches such as decentralization should be explored to meet the increasing demands of CL diagnosis and care. These could follow the multidrug-resistant tuberculosis system, where patients are diagnosed at the referral center but take treatment at lower-level health facilities closer to their homes [30].

Although the guidelines advise parasitological confirmation of patients before treatment, in practice, many patients are treated empirically. Data from a previous study in a similar population showed that this approach seems sound, as 34/40 (85%) microscopy-negative patients were confirmed by PCR [25]. Unfortunately, we could not confirm the diagnosis by PCR in this study, as samples were lost during the hospital’s occupation. Other lab tests were not routinely performed, and ECG was rarely carried out. There was one patient who died during the second treatment cycle. Since SSG is known to cause QTc interval prolongation, [31], we cannot exclude the possibility that this death was due to SSG-related QTc prolongation or arrhythmias that could have been identified by ECG. This case emphasizes the need for ECG monitoring at least before the start of treatment. Cheap and portable six-lead ECG devices attached to mobile phones were recently approved by the American Food and Drug Administration for measuring QTc intervals [32], and this could facilitate clearance for treatment with SSG at the health center or primary hospital.

This study had several limitations. Since photographs were lost due to the stolen camera, it was impossible to systematically obtain a second opinion for the outcome assessment of the patients. Therefore, outcome assessment was based solely on the opinion of the treating clinician, which is subjective, although this reflects routine practice. In future studies, we suggest that outcome assessment should be performed by two independent dermatologists, preferably in person so that flattening can be addressed properly by palpation. Second, we could not perform PCR to confirm the CL diagnosis in the majority of cases, as the slides were lost during the conflict. However, a previous study in the same population and setting showed that 92% of treated patients were confirmed by PCR. Due to similar reasons, species typing could not be carried out, although five samples from similar CL patients from this setting were confirmed to have *L. aethiopica* [25]. Third, the final outcome data could only be obtained for a small number of patients, which affects the validity of our outcome data. Although the number of patients lost to follow-up for final outcomes was very high, most patients could be reached by phone. As patient-reported outcomes were correlated with clinical outcomes, this seems to be an opportunity to supplement outcome data for future studies investigating treatment effectiveness.

## 5. Conclusions

Treatment with SSG and low-dose allopurinol led to low cure rates after one cycle, with frequent treatment extension. After 180 days, around 50% of patients were clinically cured and 30% showed improvement, while patient-reported outcomes had significantly improved. Treatment stock-outs were frequent, and bed capacity was limited, leading to long waiting lists for treatment. Increasing treatment capacity or decentralization to lower-level health facilities seems vital to meet the WHO’s NTD roadmap goals aimed to improve access to diagnosis and treatment for CL in Ethiopia.

## Figures and Tables

**Table 4:** Agreement between clinical and patient-reported outcomes.

C1	*n*/N (%)	Clear	Not Clear	Kappa Coefficient
**Cure**	*4/89 (4.5)*	1	3	0.38
**Not cured**	*85/89 (95.5)*	0	83	
		*1/87 (1.1)*	*86/87 (98.9)*	
**C2**		**Clear**	**Not Clear**	**Kappa Coefficient**
**Cure**	*2/24 (8.3)*	2	0	0.78
**Not cured**	*22/24 (91.7)*	1	20	
		*3/23 (13.0)*	*20/23 (87.0)*	
**D180**		**Clear**	**Not Clear**	**Kappa Coefficient**
**Cure**	*18/36 (50.0)*	11	5	0.69
**Not cured**	*18/36 (50.0)*	0	16	
		*11/32 (34.4)*	*21/32 (65.6)*	

## Data Availability

Data will not be made openly accessible due to ethical and privacy concerns. Data can, however, be made available after approval of a motivated and written request to ITMs Research Data Access Committee (ITMresearchdataaccess@itg.be).

## Supplementary Materials

The following supporting information can be downloaded at: https://www.mdpi.com/article/10.3390/tropicalmed8080414/s1, Figure S1: Flowchart of included patients; Table S1: Impact of cutaneous leishmaniasis on the dermatological life quality index before treatment for adults and children; Table S2: Quality of life domains affected for adults at baseline; Table S3: Quality of life domains affected for children at baseline; Table S4: Strobe checklist.
