# Supplementary material for: Treatment of Cutaneous Leishmaniasis with Sodium Stibogluconate and Allopurinol in a Routine Setting in Ethiopia: Clinical and Patient-Reported Outcomes and Operational Challenges
Source: Trop Med Infect Dis. 2023 Aug 14;8(8):414. doi: 10.3390/tropicalmed8080414 (PMC10459856; doi:10.3390/tropicalmed8080414)
Supplement: Supplementary file 1 [file tropicalmed-08-00414-s001.zip › tropicalmed-2517209-supplementary.pdf]

Supplementary Materials

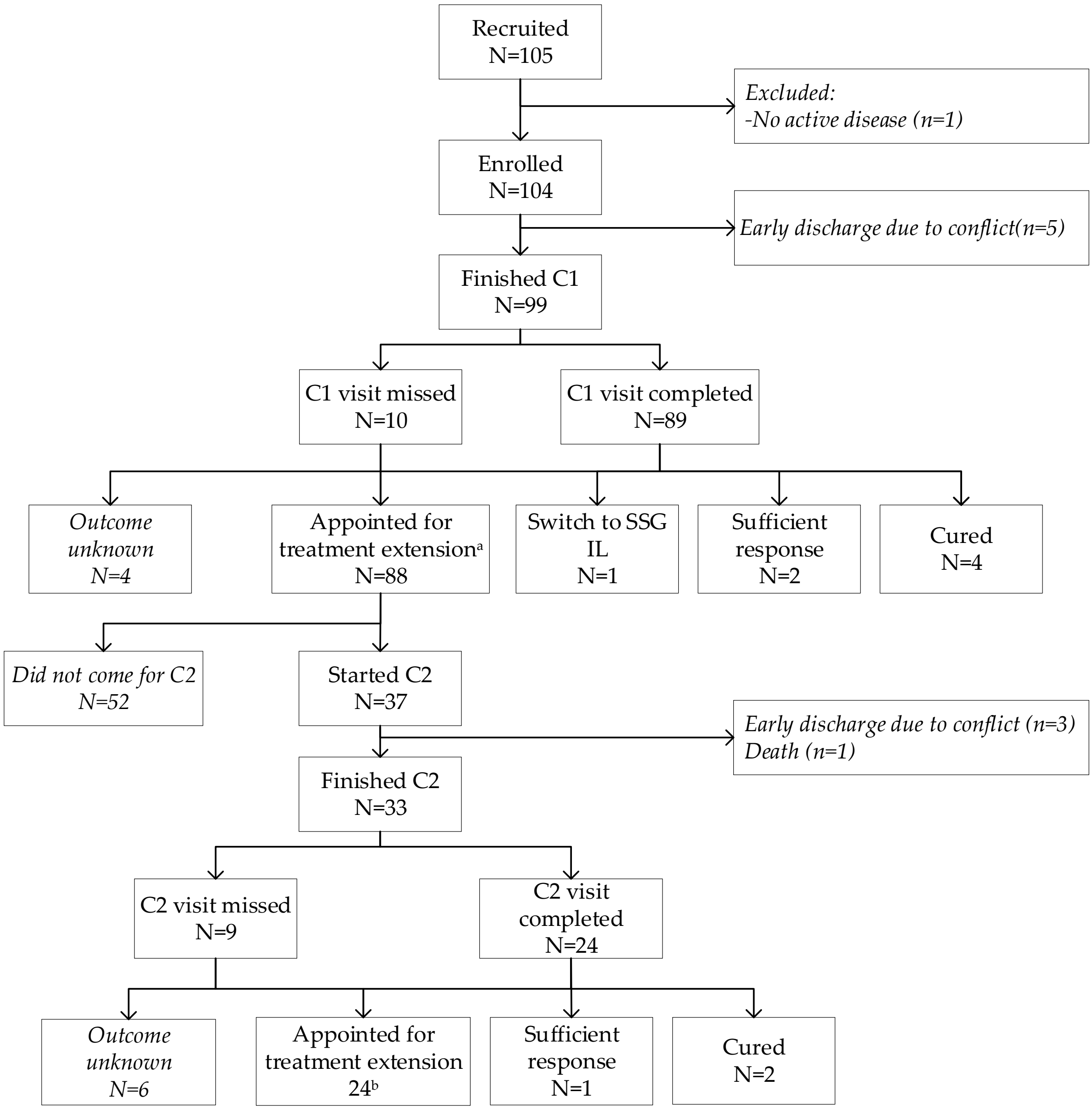

Supplementary Figure S1. Flowchart of included patients. [a]Among those extended, 82 completed C1 study visits and 6 did not. [b]Among those extended after C2, 21 completed C2 study visits and 3 did not.

Supplementary Table S1: Impact of cutaneous leishmaniasis on the dermatological life quality index before treatment for adults and children

Category	Total (%) N=99	Adults N=56	Children N=43	
No effect	6 (6.1)	0 (0)	6 (14.0)	
Small effect	26 (26.3)	11 (19.6)	15 (34.9)	
Moderate effect	22(22.2)	9 (16.1)	13 (30.2)	
Very large effect	35 (35.4)	30 (53.6)	5 (11.6)	
Extremely large effect	10 (10.1)	6 (10.7)	4 (9.3)	
Invalid	*4 (3.8)*			
Median	**10.0 (5.0 - 16.0)**	12.5 (8.0 – 18.0)	7.0 (3.0 – 11.0)	<0.001
Mean	**10.7**		7.8	

Supplementary Table S2. Quality of life domains affected for adults at baseline (n=56)

Category	No effect (0-1)	2-3 points	4-6 points	Median score
Symptoms and feelings (0-6)	5 (8.9)	27 (48.2)	24 (42.9)	3.0 (2.8 - 4.0)
Daily activities (0-6)	15 (26.8)	26 (46.4)	15 (26.8)	2.0 (1.0 -4.0)
Leisure (0-6)	21 (37.5)	25 (44.6)	10 (17.9)	2.0 (1.0 - 3.0)
School/work (0-3)	24 (42.9)	32 (57.1)	-	2.0 (0 - 3.0)
Personal relationships (0-6)	26 (46.4)	16 (28.6)	14 (25.0)	2.0 (0 - 3.2)

Supplementary Table S3. Quality of life domains affected for children at baseline (n=43)

Category	No effect (0-1)	2-3 points	4-9 points	Median score
Symptoms and feelings (0-6)	21 (48.8)	15 (34.9)	7 (16.3)	2.0 (1.0 - 3.0)
Leisure (0-9)	25 (58.1)	6 (14.0)	12 (27.9)	1.0 (0 - 4.0)
School/work (0-3)	28 (65.1)	15 (37.2)	-	1.0 (0 - 3.0)
Personal relationships (0-6)	30 (69.8)	12 (27.9)	1 (2.3)	0 (0 - 2.0)
Sleep (0-3)	38 (88.4)	5 (11.6)	-	0 (0 - 0)

Supplementary Table S4. Strobe checklist. STROBE Statement—checklist of items that should be included in reports of observational studies

	Item No	Recommendation	Manuscript section and paragraph no.
**Title and abstract**	1	(*a*) Indicate the study's design with a commonly used term in the title or the abstract	Abstract paragraph 2
		(*b*) Provide in the abstract an informative and balanced summary of what was done and what was found	Abstract paragraph 2 and 3
**Introduction**			
Background/rationale	2	Explain the scientific background and rationale for the investigation being reported	Introduction paragraph 2-4
Objectives	3	State specific objectives, including any prespecified hypotheses	Introduction paragraph 5
**Methods**			
Study design	4	Present key elements of study design early in the paper	Methods: design, population, recruitment and sample size: paragraph 1
Setting	5	Describe the setting, locations, and relevant dates, including periods of recruitment, exposure, follow-up, and data collection	Methods: design, population, recruitment and sample size: paragraph 1
Participants	6	(*a*) *Cohort study*—Give the eligibility criteria, and the sources and methods of selection of participants. Describe methods of follow-up<br>*Case-control study*—Give the eligibility criteria, and the sources and methods of case ascertainment and control selection. Give the rationale for the choice of cases and controls<br>*Cross-sectional study*—Give the eligibility criteria, and the sources and methods of selection of participants	Methods: design, population, recruitment and sample size: paragraph 1,2<br>Methods: clinical outcome assessment paragraph 1-2<br>Methods: patient tracing
		(*b*) *Cohort study*—For matched studies, give matching criteria and number of exposed and unexposed<br>*Case-control study*—For matched studies, give matching criteria and the number of controls per case	
Variables	7	Clearly define all outcomes, exposures, predictors, potential confounders, and effect modifiers. Give diagnostic criteria, if applicable	Methods: Clinical outcome assessment<br>Methods: patient-reported outcome measures
Data sources/ measurement	8*	For each variable of interest, give sources of data and details of methods of assessment (measurement). Describe comparability of assessment methods if there is more than one group	Methods: Clinical outcome assessment<br>Methods: patient-reported outcome measures
Bias	9	Describe any efforts to address potential sources of bias	Methods: patient tracing

Study size	10	Explain how the study size was arrived at	Methods: design, population, recruitment and sample size: paragraph 3
Quantitative variables	11	Explain how quantitative variables were handled in the analyses. If applicable, describe which groupings were chosen and why	Methods: patient-reported outcome measures<br>Other variables are kept as quantitative
Statistical methods	12	(*a*) Describe all statistical methods, including those used to control for confounding	Methods: data collection and analysis
		(*b*) Describe any methods used to examine subgroups and interactions	NA
		(*c*) Explain how missing data were addressed	Missing data is described in tables as separate category.
		(*d*) *Cohort study*—If applicable, explain how loss to follow-up was addressed<br>*Case-control study*—If applicable, explain how matching of cases and controls was addressed<br>*Cross-sectional study*—If applicable, describe analytical methods taking account of sampling strategy	Methods: patient tracing
		(*e*) Describe any sensitivity analyses	
**Results**			
Participants	13*	(a) Report numbers of individuals at each stage of study—eg numbers potentially eligible, examined for eligibility, confirmed eligible, included in the study, completing follow-up, and analysed	Supplementary figure 1
		(b) Give reasons for non-participation at each stage	
		(c) Consider use of a flow diagram	
Descriptive data	14*	(a) Give characteristics of study participants (eg demographic, clinical, social) and information on exposures and potential confounders	Table 1
		(b) Indicate number of participants with missing data for each variable of interest	Table 1-3
		(c) *Cohort study*—Summarise follow-up time (eg, average and total amount)	Results: treatment and follow-up
Outcome data	15*	*Cohort study*—Report numbers of outcome events or summary measures over time	Results: treatment and follow-up, Table 3
		Case-control study—Report numbers in each exposure category, or summary measures of exposure	
		Cross-sectional study—Report numbers of outcome events or summary measures	
Main results	16	(*a*) Give unadjusted estimates and, if applicable, confounder-adjusted estimates and their precision (eg, 95% confidence interval). Make clear which confounders were adjusted for and why they were included	Table 2-3

		(*b*) Report category boundaries when continuous variables were categorized	NA
		(*c*) If relevant, consider translating estimates of relative risk into absolute risk for a meaningful time period	
Other analyses	17	Report other analyses done—eg analyses of subgroups and interactions, and sensitivity analyses	NA
**Discussion**			
Key results	18	Summarise key results with reference to study objectives	Discussion: paragraph 1
Limitations	19	Discuss limitations of the study, taking into account sources of potential bias or imprecision. Discuss both direction and magnitude of any potential bias	Discussion: paragraph 10
Interpretation	20	Give a cautious overall interpretation of results considering objectives, limitations, multiplicity of analyses, results from similar studies, and other relevant evidence	Discussion
Generalisability	21	Discuss the generalisability (external validity) of the study results	NA
**Other information**			
Funding	22	Give the source of funding and the role of the funders for the present study and, if applicable, for the original study on which the present article is based	Funding statement

*Give information separately for cases and controls in case-control studies and, if applicable, for exposed and unexposed groups in cohort and cross-sectional studies.

Note: An Explanation and Elaboration article discusses each checklist item and gives methodological background and published examples of transparent reporting. The STROBE checklist is best used in conjunction with this article (freely available on the Web sites of PLoS Medicine at http://www.plosmedicine.org/, Annals of Internal Medicine at http://www.annals.org/, and Epidemiology at http://www.epidem.com/). Information on the STROBE Initiative is available at www.strobe-statement.org.
